# Effects of Ultramicronized Palmitoylethanolamide (um-PEA) in COVID-19 Early Stages: A Case–Control Study

**DOI:** 10.3390/ph15020253

**Published:** 2022-02-19

**Authors:** Maria Albanese, Giulia Marrone, Agostino Paolino, Manuela Di Lauro, Francesca Di Daniele, Carlo Chiaramonte, Cartesio D’Agostini, Annalisa Romani, Alessandro Cavaliere, Cristina Guerriero, Andrea Magrini, Nicola Biagio Mercuri, Nicola Di Daniele, Annalisa Noce

**Affiliations:** 1 Neurology Unit, Department of Systems Medicine, University of Rome Tor Vergata, 00133 Rome, Italy; maria.albanese@ptvonline.it (M.A.); mercurin@med.uniroma2.it (N.B.M.); 2 UOC of Internal Medicine-Center of Hypertension and Nephrology Unit, Department of Systems Medicine, University of Rome Tor Vergata, 00133 Rome, Italy; dilauromanuela@gmail.com (M.D.L.); cristina.guerriero@alumni.uniroma2.eu (C.G.); didaniele@med.uniroma2.it (N.D.D.); 3 Department of Biomedicine and Prevention, University of Rome Tor Vergata, 00133 Rome, Italy; agostino.paolino.pv@gmail.com (A.P.); andrea.magrini@uniroma2.it (A.M.); 4 PhD School of Applied Medical, Surgical Sciences, University of Rome Tor Vergata, 00133 Rome, Italy; francesca.didaniele@gmail.com; 5 UOSD of Dermatology, Department of Systems Medicine, University of Rome Tor Vergata, 00133 Rome, Italy; 6 Department of Statistics, University of Rome Tor Vergata, 00133 Rome, Italy; chiaramonte.carlo43@gmail.com; 7 Department of Experimental Medicine, University of Rome Tor Vergata, 00133 Rome, Italy; cartesio.dagostini@ptvonline.it; 8 Laboratory of Clinical Microbiology, Policlinico Tor Vergata, 00133 Rome, Italy; 9 PHYTOLAB (Pharmaceutical, Cosmetic, Food Supplement, Technology and Analysis), DiSIA, University of Florence, Sesto Fiorentino, 50019 Florence, Italy; 10 Clinic Department of Gynecology Fabia Mater, 00171 Rome, Italy; alessandrocavaliere01@gmail.com; 11 IRCCS Santa Lucia Foundation, 00179 Rome, Italy

**Keywords:** SARS-CoV-2, COVID-19, um-PEA, oral food supplement, cytokines storm, oxidative stress, long COVID syndrome, adjuvant treatment

## Abstract

Ultramicronized palmitoylethanolamide (um-PEA), a compound with antioxidant, anti-inflammatory and neuroprotective properties, appears to be a potential adjuvant treatment for early stages of Coronavirus disease 2019 (COVID-19). In our study, we enrolled 90 patients with confirmed diagnosis of COVID-19 that were randomized into two groups, homogeneous for age, gender and BMI. The first group received oral supplementation based on um-PEA at a dose of 1800 mg/day for a total of 28 days; the second group was the control group (R.S. 73.20). At baseline (T0) and after 28 days of um-PEA treatment (T1), we monitored: routine laboratory parameters, inflammatory and oxidative stress (OS) biomarkers, lymphocytes subpopulation and COVID-19 serological response. At T1, the um-PEA-treated group presented a significant reduction in inflammation compared to the control group (CRP *p* = 0.007; IL-6 *p* = 0.0001; neutrophils to lymphocytes ratio *p* = 0.044). At T1, the controls showed a significant increase in OS compared to the treated group (FORT *p* = 0.05). At T1, the um-PEA group exhibited a significant decrease in D-dimer levels (*p* = 0.0001) and higher levels of IgG against SARS-CoV-2 (*p* = 0.0001) compared to the controls. Our data demonstrated, in a randomized clinical trial, the beneficial effects of um-PEA in both asymptomatic and mild-symptomatic patients related to reductions in inflammatory state, OS and coagulative cascade alterations.

## 1. Introduction

In late 2019, a new kind of microorganism began to spread from the city of Wuhan in China. Later, a different member of the Coronaviridae family, a beta-coronavirus called Severe Acute Respiratory Syndrome Coronavirus-2 (SARS-CoV-2), was identified as the leading cause of this new syndrome, named COVID-19 (Coronavirus disease 2019).

Coronaviruses, identified in the mid-1960s, are known to infect humans as well as other animals; the epithelial cells of the respiratory and gastrointestinal tracts are considered to be their primary target cells [1]. However, it is difficult to think that the penetration of the viral agent into the organism remains so limited. In fact, there is enormous variability regarding the clinical manifestations of COVID-19: general symptoms (fever, muscle aches, asthenia), pulmonary damage (cough, “shortness of breath”, acute respiratory syndrome) [2], cardiac involvement (acute heart failure, myocarditis, shock), gastrointestinal symptoms (diarrhea, nausea, vomiting, and abdominal pain) [3], liver [4] and kidney damage [5], neurological damage [6], and cutaneous alterations such as rash and skin lesions [7]. Preclinical and clinical evidence suggest that Coronaviruses may have increased tissue invasiveness and evident neurotropism, which could lead to much more complex situations [8].

During viral infection, the organism activates a defensive neuroinflammatory process through cells of the innate immune system; under certain conditions, this process can become pathological, leading to a dysregulation of the immune system itself. This process may become uncontrollable due to an abnormal massive release of pro-inflammatory cytokines by non-neuronal cells belonging to the immune system, such as mast cells in the periphery, and microglial cells in the central nervous system (CNS). The activity of this kind of cytokines worsens the neuroinflammatory state and contributes to inducing multifactorial pathogenesis of the disease [9].

In this case, SARS-CoV-2 infection is characterized by a systemic inflammatory storm, with a massive release of interleukins (ILs), such as IL-6 and tumor necrosis factor (TNF)-α, chemokines and other mediators of inflammation, which lead to the rupture of the blood–brain barrier (BBB); it is also responsible for worsening of the ongoing neuroinflammatory process [10]. The damage of the BBB allows ILs to activate Toll-like receptors (TLRs) located on the microglia [11] that, in turn, activate astrocytes, monocytes, dendritic cells, and white blood cells that have already invaded the CNS, feeding the neuroinflammatory process [12], as highlighted by the hypertrophy of glial cells and neuronal apoptosis [13]. Even clinically mild respiratory disorders are characterized by important spreading of inflammatory messengers, which could impair the BBB [14].

The current pandemic caused by SARS-CoV-2 has rekindled attention to the neurovirulence of this virus and to the possible direct involvement of the nervous system in some patients. Psychiatric and neurological complications were reported during the SARS epidemic in 2003, characterized by isolated cases of fatal encephalomyelitis from Coronavirus OC43, although there was poor lung involvement [15].

As mortality rates (COVID-related deaths) vary from 1% to 7%, COVID-19 patients may suffer from a multitude of long-term health consequences still to be evaluated. Potential long-term manifestations may affect the CNS, heart, lungs, the hematopoietic system, kidneys and the gastrointestinal system, including psychosocial disorders, as well as post-intensive care syndrome for those patients who were hospitalized [16].

Among patients admitted to the intensive care unit (ICU), cognitive and psychophysical impairments (including muscular mass loss, weakness, diaphragm dysfunction, anxiety and depression) might manifest and gradually worsen.

On the other hand, even mild symptomatic patients may experience persistent symptoms, from weeks to months [17]. Recent studies suggest residual symptoms of SARS-CoV-2 infection, including dyspnea, fatigue, chest pain, cognitive effects, arthralgia and a decline in quality of life [18]. The most common long-term features include chronic fatigue syndrome and a variety of neurological involvement-based disorders, such as loss of smell and taste; they usually last 1 or 2 months after infection, but occasionally, it may take several months to completely recover [19]. Depression and migraine-like headaches have also been reported at different levels of severity [20]. The severity of an acute illness can depend on the presence of sleep disorders [21], palpitations and chest pain, dermatologic involvement [22], pulmonary symptoms (such as a reduction in diffusion capacity), radiological features, and shortened resistance to efforts [23,24].

The current therapy, which is mostly used to counteract the symptoms of infection, is based on anti-inflammatory and immunomodulating products and on antiviral actions observed in in vitro studies. These drugs are not specifically for the treatment of COVID-19 and they also present several limitations [25]. For this reason, in addition to traditional drugs, it should be considered that a possible adjuvant treatment may have an important role, especially those belonging to the natural bioactive compounds (NBCs) family. There are currently several studies investigating the possible role of oral food supplements based on NBCs in alleviating COVID-19 symptoms. Among these, a very interesting one is palmitoylethanolamide (PEA), an endogenous molecule belonging to the *N*-acyl-ethanolamine family with anti-inflammatory properties. PEA is synthesized “on demand” in cases of “stress factors” that cause inflammation so as to restore tissue homeostasis, acting on the regulation of non-neuronal cells [26,27].

A growing body of evidence suggests that PEA acts on several molecular and cellular mechanisms, both in vitro and in vivo. One of these includes an autacoid role in controlling mast cells’ activities in inflammatory response [28]. In particular, it has been shown that PEA levels increase with stressors provoking factors, especially inflammation, thanks to the production/activation from mast cells and microglia. PEA has also shown to be significantly active on the cannabinoid CB2-receptor, inducing a peripheral antinociceptive effect [29], in addition to prevention of IκB-α degradation and p65 nuclear factor-κB nuclear translocation [30]. Moreover, the role of PEA was demonstrated in increasing the expression of glial IL-10 [31].

Furthermore, PEA may be considered a natural disease-modifying agent instead of a symptoms-reducing one, as it modulates the inflammatory processes and immune system. When PEA endogenous synthesis is low, PEA exogenous supplementation improves the molecule’s effectiveness [32]. Many studies have shown its pivotal role in several downregulating mechanisms dealing with pain modulation [33], hyperalgesia [34], edema formation [35], activation of C-fibers [36], sensory neuropeptides release [37], and activation of IL1-β in preclinical tests [38].

In the last few decades, several clinical studies have highlighted the important role of PEA and its ultramicronized form in many pathological conditions, such as Parkinson’s [39], carpal tunnel syndrome with neuropathic pain and altered sleep–wake rhythm [40], fibromyalgia [41], delirium manifestations [42] and in Alzheimer’s disease with reduced neuronal trophic support [43]. 

Chemically, it is noteworthy that PEA is a highly lipophilic compound. Considering that the low dissolution rate of poorly water-soluble drugs in biological fluids is the limiting step in their absorption, and as a consequence, in their pharmacological activity, PEA undergoes different metabolic pathways in order to improve its availability. The size of natural PEA is about 100–700 μm, while its micronized (m-PEA) form is 6–10 μm and its ultramicronized form (um-PEA) contains particles between 1 and 10 μm. The smaller particle size (with higher surface-to-volume ratio) contributes to better solubility and, thus, to a better distribution in tissues and a higher biological efficacy [32,44,45].

The aim of our study is to evaluate the potential beneficial effects of micronized and ultramicronized palmitoylethanolamide (m-PEA + um-PEA) administration as an add-on to standard therapy at a dose of 1800 mg/day for 28 days in a group of COVID-19 patients, compared to a group of patients treated only with standard therapy.

## 2. Results

The clinical features of all enrolled patients are reported in Table 1. The two groups of the study—population A (um-PEA-treated patients) and B (control group)—were homogeneous for age, gender and body mass index (BMI).

**Figure 1:**
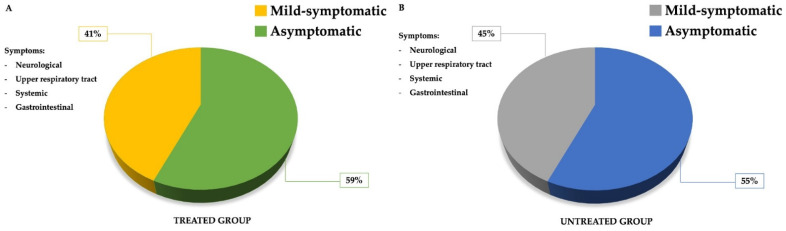
Frequency and type of symptoms at baseline (T0) in treated group (panel **A**) and in untreated group (panel **B**).

At T0, among the treated group, 41% were mild-symptomatic and 59% were asymptomatic (Figure 1, panel A), while in the untreated group, 45% presented mild symptoms and the remaining 55% were asymptomatic (Figure 1, panel B). 

The most frequent symptoms were neurological, followed by upper respiratory tract, systemic and gastrointestinal symptoms. Among the neurological manifestations, most patients presented anosmia, ageusia, muscular aches and headache. The upper respiratory tract manifestations were represented by dry cough, dyspnea and sore throat. Systemic symptoms were represented by fever > 37.5 °C and by asthenia. Gastrointestinal symptoms were rare and consisted of diarrhea and nausea.

Regarding oxidative stress (OS), monitored by the free oxygen radical defense (FORD) test and the free oxygen radicals test (FORT), we observed an increase in FORD test score in the treated group compared to the control group, but this enhancement was not statistically significant (Table 2). Conversely, FORT showed a significant increase between T0 versus T1 in the control group (*p* = 0.05). 

Concerning white blood cells, we analyzed the impact of um-PEA administration on lymphocytes as absolute values and highlighted their prompt increase in the treated group (*p* = 0.02), as well as in the CD3+CD8+ lymphocyte subpopulation as absolute counts. 

We also evaluated the impact of um-PEA on inflammatory status, showing a significant reduction in the treated group for neutrophil-to-lymphocyte ratio (*p* = 0.044), C-reactive protein (CRP) (*p* = 0.007) and IL-6 (*p* = 0.0001) at T1. For other inflammatory biomarkers, we observed a higher reduction in TNF-α in the um-PEA-group, though this did not reach statistical significance.

Moreover, we noticed a significant reduction in D-Dimer in treated patients compared to the control group (*p* = 0.0001). At the end of treatment, we found higher levels of IgG against SARS-CoV-2 in the treated group compared to controls.

## 3. Discussion

Currently, there is no standardized therapeutic treatment able to counteract progression versus severe pictures of SARS-CoV-2 infection and the development of long-COVID syndrome. For this reason, it is important to identify a new adjuvant therapeutic approach useful for managing COVID-19.

In the pathogenesis of COVID-19, the “cytokine storm” (CS) plays a pivotal role. The latter is characterized by high serum levels of pro-inflammatory biomarkers (such as CRP, IL-6, etc.) and by an uncontrolled inflammatory process that, if not inhibited, induces multi-organ failure [46,47,48]. Therefore, the significant reduction in inflammatory status that we observed in our um-PEA-treated patients seems to contrast the onset of the systemic inflammation and the CS. In fact, in our study, we found a significant decrease in CRP and IL-6 after 28 days of um-PEA assumption in the treated group. We also observed a trend of TNF-α reduction in the same group, though this was not statistically significant. 

Another important result that we noticed in our study was the significant increase in OS in the control group at T1. It is well-known that several interstitial viruses, including SARS-CoV-2, cause an enhancement in oxidized biomolecules such as DNA, lipids, and proteins. In fact, they are able to induce a high production of reactive oxygen species (ROS), triggering and amplifying a dysregulated immune response [49]. Moreover, these viruses can cause an upregulated nitric oxide synthase-2 (NOS2) expression. In view of the obtained results, we speculate that um-PEA may be useful in OS and inflammation reduction, as it seems to be able to decrease ROS production and, at the same time, positively modulate the release of pro-inflammatory cytokines, mitigating the clinical symptoms [50,51]. Furthermore, in the um-PEA-treated group, we highlighted an increased trend of FORD, namely the antioxidant defenses. During SAR-CoV-2 infection, the amplified ROS production induces a consequent reduction in human antioxidant defenses with an increased susceptibility to severe pictures of the disease, especially in elderly patients and in those affected by chronic-degenerative non-communicable diseases. Therefore, the antioxidant properties of um-PEA could attenuate COVID-19 complications and tissue damage in several organs and systems. 

We also found a significant enhancement of lymphocytes, in absolute values, and a significant reduction in neutrophil-to-lymphocytes ratio in the um-PEA-treated group. This index is a negative prognostic parameter in patients with sepsis [52] and is an indicator of systemic inflammation [53]. The neutrophil-to-lymphocytes ratio, which is easily available, has recently been proposed and has been recognized as an independent predictor for several pathological conditions such as cancer, cardiovascular diseases, etc. In particular, an increase in this index has been associated with higher mortality [54] in hospitalized COVID-19 patients [55]. Normal values for the neutrophil-to-lymphocytes ratio, in healthy adult subjects, are between 1.0 and 2.3 [55]. In detail, in our study, we noted a normalization of this ratio only in the um-PEA-treated group. In this context, we assumed that um-PEA might be a promising agent in reducing the local translocation and the migration of neutrophils at the site of infection, thus contributing to limiting the immune and inflammatory response.

In the treated group of patients, we also noted a significant increase in CD3+CD8+ lymphocyte subpopulation at T1. We speculated that this elevation may be correlated to the um-PEA treatment, as the drug seems to prevent the consumption of T-cells and the worsening of COVID-19 versus more severe clinical manifestations. In accordance with previous studies [56], our data confirm that um-PEA is able to directly modulate T-cells response by the regulation of inflammatory pathways.

Another parameter related to poor survival in COVID-19 patients is represented by D-dimer levels. The enhancement of this biomarker, indicative of activation of coagulation pathways and of thrombosis, has been associated with unfortunate prognosis in the early stages of COVID-19 patients [57]. Interestingly, in our study, the oral assumption of um-PEA seems to counteract this increase, which is commonly found in COVID-19 clinical evolution, suggesting that this compound could display a protective effect against vascular damage.

Finally, we observed a prompt and better response of the immune system in the treated group compared to the control one, tested by total antibodies against SARS-CoV-2. In fact, in these patients, we appreciated higher antibody titers compared to the controls.

The presence of antibodies against SARS-CoV-2 may be the best indicator of protection against reinfection. This finding reinforces the concept that um-PEA is able to exert an immunomodulatory action as an agonist, binding to peroxisome proliferator-activated receptor α (PPARα), which is expressed in different tissues, especially in the immune cells such as B-lymphocytes [46,58,59].

### Limitations of the Study

The absence of a placebo group is a limitation for our study, as it is minimized by the law of statistical regression towards the average. Moreover, it is well known that the inclusion of a placebo group would mean a deviation from the “principle of pragmatism”. In fact, pragmatic trials are designed to test how a heterogeneous population of patients responds to interventions under the closest approximation of conditions found in clinical practice, namely, in real life. 

## 4. Materials and Methods

### 4.1. Patients and Enrollment Criteria

From September 2020 to January 2021, a total of 90 patients with confirmed COVID-19 from reverse transcriptase real time (rRT)-polymerase chain reaction (PCR) naso-oropharyngeal swab were enrolled and they signed a written informed consent to participate in the study. The study protocol complied with the Declaration of Helsinki and was approved on 21 May 2020 by the Ethical Committee of Fondazione Policlinico Tor Vergata (PTV) of Rome (R.S. 73.20). Inclusion criteria were: age comprised between 18 and 80 years, both sexes, confirmed virological COVID-19 within 24 h with no symptoms or mild-to-moderate infection (the latter presenting one or more of the following symptoms: fever > 37.5 °C, cough, headache, asthenia, anosmia, diarrhea, SpO2 > 93% or PaO2/FiO2 > 300 mmHg without oxygen inhalation) [25]. Exclusion criteria were: pregnancy and breastfeeding, non-acceptance of the informed consent and data processing, subjects with severe respiratory failure who require an invasive mechanic ventilation, subjects with allergy or hypersensitivity to the PEA or to one or more of its excipients. 

Joining the study included a complete medical history and comorbidities in order to gather information about general health status, current medications and the most common symptoms related to COVID-19 (anosmia, ageusia, headache, fever, asthenia, cough, muscle aches, tiredness and gastrointestinal symptoms) at both timepoints of the study (T0, enrollment and T1, after 28 days).

### 4.2. m-PEA + um-PEA Treatment

All enrolled patients were in home-based isolation and were randomized into two groups, according to the study protocol: the first group (45 patients) was treated with standard therapy and an add-on oral treatment in microgranules for sublingual use (Normast MPS^®^) and the second group (45 patients) was treated only with standard therapy, represented as the control group.

The first group consumed 2 sticks of PEA m + PEA-um, a food for special medical purposes, (Normast MPS^®^ microgranules for sublingual administration Epitech group SpA—Saccolongo-PD) per day (morning and evening) at a dose of 1800 mg/day for 28 days.

The batch number of the Normast MPS^®^ administered is 19A517; the expiry date is 11/2022, and a description of its active ingredients and excipients is reported Table 3.

### 4.3. Statistical Analysis 

All data were entered into an Excel spreadsheet (Microsoft, Redmond, WA, USA) and the analysis was performed using the Windows Social Science Statistics Package, version 25.0 (IBM_SPSS, Chicago, IL, USA).

The descriptive statistics consider the mean ± standard deviation for the parameters with normal distribution (after confirmation with histograms and the Kolmogorov–Smirnov test), while for the non-normal variables, we consider the median and the interval (minimum:maximum). All parameters detected at baseline (T0) and T1 were compared using a *t*-test for normal variables, while for non-normal variables, we conducted a Mann–Whitney test. A *p*-value < 0.05 was considered statistically significant. 

### 4.4. Laboratory Parameters 

At T0 and T1, all enrolled patients underwent the following laboratory exams: complete blood count, erythrocyte sedimentation rate (ESR), biomarkers of liver and renal function, muscle damage indices, vitamin D, complete blood clotting test, CRP, IL-6, TNF-α, and lymphocyte subpopulations. Moreover, we calculated inflammatory indices such as: platelets-to-lymphocytes, neutrophils-to-lymphocytes and lymphocytes-to-monocytes ratios. At T1, we also evaluated COVID-19 serological response.

Complete blood count was determined by an automated method (Dasit-Sysmex, Milan, Italy); ESR with the fully automated analyzer test-1 (Alifax Srl, Polverara, Padova, Italy); and CRP was analyzed by the immunoturbidimetric method (Abbott Diagnostics, Milan, Italy). Routine laboratory parameters were evaluated by Abbott Architect Instrument (Abbott Diagnostics Milan, Italy).

Muscle damage indices (Creatine kinase-CPK and myoglobin) were evaluated using chemiluminescence immunoassays with Abbott Architect Instrument (Abbott Diagnostics, Milan, Italy).

Total serum vitamin D was measured by electrochemiluminescence (Abbott Architect Instrument, Milan, Italy). Blood clotting tests were detected by ACLTOP (Werfen, Milan, Italy). Serum levels of IL-6 were measured by chemiluminescence (IMMULITE 2000, Siemens, Milan, Italy); TNF-α levels were measured by the ELISA technique (DRG, International Instruments GmbH, Marburg, Germany).

Lymphocyte subpopulations were assessed by flow cytometric analysis, performed on whole blood samples, obtained from venous sampling. Samples were incubated with fluorescent monoclonal antibodies (mAbs) and using FACSCanto II with two lasers and up to 6–7 colors (BD, Biosciences, San Jose, CA, USA) and FACSDiva Software (BD, Biosciences, San Jose, CA, USA) for acquisition and analysis. The samples were first incubated with mAbs and then processed with red cell lysing (using 1× ammonium chloride solution; BD, Bioscience, San Jose, CA).

Moreover, all patients also underwent to capillary sampling, using the CR4000 tool for OS evaluation and for the assessment of antioxidant defense mechanisms. In particular, a FORT and a FORD test were performed. The first test detects the levels of circulating oxygen free radicals and the second test indirectly determines blood antioxidant defenses.

The SARS-CoV-2 IgG assay is able to detect IgG antibodies directed against the SARS-CoV-2 nucleocapsid protein in serum and plasma using chemiluminescent microparticle capture immunoassay (CMIA) technology (Abbott, IL, USA). The anti-SARS-CoV-2 IgG antibodies in the sample bind the microparticles coated with SARS-CoV-2 antigens. The resulting chemiluminescent reaction is measured in relative light units (RLU). There is a direct link between the amount of anti-SARS-CoV-2 IgG antibodies in the sample and the RLUs measured by the optical system. This link is reflected in the calculated index (S/C). The standard unit of measure for results for the SARS-CoV-2 IgG assay is index (S/C) and the cutoff is 1.4 index (S/C). All parameters were analyzed according to standard procedures in the Clinical Chemical Laboratories of the University Hospital, PTV of Rome.

## 5. Conclusions

um-PEA oral supplementation could potentially be an adjuvant treatment able to counteract COVID-19 clinical manifestations thanks to its anti-inflammatory, antioxidant, immunostimulatory and neuroprotective properties. This oral food supplement appears to be safe and well-tolerable in the early stages of COVID-19, as shown in our study population.

Moreover, our data demonstrated the beneficial effects of um-PEA in a population of asymptomatic and mild-symptomatic patients related to a significant reduction in inflammatory state and OS and to an alteration of the coagulative cascade, for the first time in a randomized clinical trial. At the same time, um-PEA appears to play a pivotal role in immune response. Further randomized clinical trials in a larger population will be required to confirm these data and to evaluate the possible effects of um-PEA on long COVID syndrome.

## Figures and Tables

**Table 1:** Clinical features of enrolled patients. The values are expressed as mean ± standard deviation; *p* < 0.05 is considered statistically significant; n.s. = statistically not significant.

Parameters	Cases (*n* = 45)	Controls (*n* = 45)	*p-*Value
Age (years)	45.6 ± 13.7	55.8 ± 22.5	n.s.
Male/female (*n*)	17/28	22/23	n.s.
Weight (kg)	69.3 ± 6.9	70.4 ± 7.1	n.s.
BMI (kg/m^2^)	24.4 ± 3.4	25.6 ± 5.8	n.s.

BMI: body mass index.

**Table 2:** Main results observed in the case and control groups. The values are expressed as mean ± standard deviation; *p* < 0.05 is considered statistically significant; n.s. = statistically not significant.

Parameters	Cases (*n* = 45)		Controls (*n* = 45)		*p-*Value
	T0 (Mean ± SD)	T1 (Mean ± SD)	T0 (Mean ± SD)	T1 (Mean ± SD)	
Red blood cell (10^4^/µL)	4.88 ± 0.52	4.64 ± 0.53	4.30 ± 0.78	4.21 ± 0.69	n.s.
Hemoglobin (g/dL)	13.98 ± 1.82	13.35 ± 1.74	12.54 ± 2.27	12.4 ± 2.00	n.s.
Hematocrit (%)	41.9 ± 5.2	40.1 ± 6.62	36.9 ± 8.08	37.3 ± 5.34	n.s
MCV (fL)	86.1 ± 6.1	86.7 ± 6.08	87.9 ± 5.96	88.3 ± 5.02	n.s.
Neutrophil (10^3^/µL)	3.44 ± 2.04	3.4 ± 2.02	4.56 ± 2.60	4.43 ± 2.34	n.s.
Lymphocytes (10^3^/µL)	1.64 ± 0.60	1.96 ± 0.53	1.32 ± 0.56	1.62 ± 0.74	0.02
Neutrophil-to-Lymphocyte ratio	2.43 ± 2.03	1.78 ± 3.8	3.45 ± 4.64	2.7 ± 3.16	0.04
Platelets (10^3^/µL)	241.69 ± 74.7	267.8 ± 43.7	239.63 ± 95.4	240.75 ± 96.7	n.s.
Myoglobin (mg/mL)	43.02 ± 53.8	38.28 ± 45.2	110.75 ± 154.7	77.26 ± 93.3	n.s.
D-Dimer (ng/mL)	686.19 ± 1348.77	366.07 ± 230.76	1032 ± 1258.42	670.57 ± 507.08	0.0001
PT (%)	94.64 ± 18.62	97.35 ± 20.16	81.61 ± 15.76	88.40 ± 17.31	n.s.
PT (INR)	1.09 ± 0.15	1.32 ± 1.74	1.13 ± 0.14	1.08 ± 0.12	n.s.
PT (s)	13.41 ± 3.93	12.06 ± 2.05	13.83 ± 1.91	13.18 ± 1.72	n.s.
Fibrinogen (mg/dL)	298.5 ± 93.7	260.83 ± 100.0	455.14 ± 223.13	375.14 ± 167.3	n.s.
Antithrombin III (%)	107.25 ± 6.95	102.5 ± 4.43	98.54 ± 17.8	119.65 ± 123.4	n.s.
Creatininemia (mg/dL)	0.83 ± 0.19	0.87 ± 0.25	0.98 ± 0.84	0.95 ± 0.88	n.s.
GFR (mL/min)	87.56 ± 17.83	86.02 ± 19.82	94.81 ± 37.14	98.12 ± 35.12	n.s.
Azotemia (md/dL)	30.47 ± 8.44	32.44 ± 14.58	36.22 ± 16.54	35.16 ± 20.95	n.s.
Vitamin D (ng/mL)	32.0 ± 16.03	30.16 ± 15.41	21.57 ± 10.12	25.54 ± 13.20	n.s.
ESR (mm/h)	24.04 ± 20.37	14.89 ± 11.65	22.30 ± 17.60	15.90 ± 12.46	n.s.
CRP (mg/dL)	7.20 ± 12.95	1.55 ± 1.80	20.03 ± 24.59	9.79 ± 16.90	0.007
TNF-α (pg/mL)	19.23 ± 20.07	8.15 ± 8.69	50.00 ± 114.20	31.84 ± 84.69	n.s.
IL-6 (pg/mL)	11.22 ± 19.58	3.30 ± 1.54	24.20 ± 23.00	15.36 ± 19.90	0.0001
GOT/AST (U/L)	29.21 ± 10.94	24.7 ± 6.93	28.13 ± 14.76	25.11 ± 10.84	n.s.
GPT/ALT (U/L)	31.72 ± 20.09	26.82 ± 12.91	23.38 ± 15.22	21.0 ± 10.9	n.s.
γ -GT (U/L)	27.83 ± 38.7	20.55 ± 12.32	33.35 ± 44.6	24.86 ± 18.85	n.s.
Creatine kinase (U/L)	82.46 ± 57.65	92.46 ± 43.53	93.0 ± 127.57	56.59 ± 45.59	n.s.
FORD (mmol/L Trolox)	1.04 ± 0.34	1.49 ± 0.43	1.09 ± 0.35	1.23 ± 0.32	n.s.
FORT (U)	271.07 ± 156.82	222.02 ± 107.71	229.90 ± 143.98	283.30 ± 111.08	0.05
CD3 + CD8 + absolute count	526.98 ± 330.71	636.41 ± 325.26	497.13 ± 237.31	487.60 ± 196.81	0.0001
Anti-SARS-CoV-2 IgG	NA	4.37 ± 1.62	NA	2.89 ± 2.03	0.0001

CRP: C-reactive protein; ESR: erythrocyte sedimentation rate; FORD: Free Oxygen Radical Defense; FORT: Free Oxygen Radicals Test; GFR, Glomerular Filtration Rate; IL, interleukin; MCV, mean corpuscular volume; NA, not available; PT, prothrombin time; TNF, tumor necrosis factor.

**Table 3:** Description of active ingredients and excipients of Normast MPS^®^.

Composition NORMAST^®^ MPS Microgranules	%
Ultramicronized Palmitoylethanolamide (um-PEA, 600 mg)	48.48
Micronized Palmitoylethanolamide (m-PEA,300 mg)	24.24
Fructose	15.15
Sorbitol	9.33
Polysorbate 80	0.36
Palmitic esters of sucrose	1.45
Cross-linked sodium carboxymethylcellulose	0.97 TOTAL 100.00

## Data Availability

Data is contained within the article.

## Abbreviations

BBB	Blood–brain barrier
BMI	Body mass index
CNS	Central nervous system
CPK	Creatine kinase
COVID-19	Coronavirus disease-19
CRP	C-reactive protein
CS	Cytokine storm
ESR	Erythrocyte sedimentation rate
FORD	Free oxygen radical defense
FORT	Free oxygen radicals test
ICU	Intensive care unit
IL	Interleukin
mAbs	Monoclonal antibodies
NBCs	Natural bioactive compounds
NOS2	Nitric oxide synthase-2
OS	Oxidative stress
PCR	Polymerase chain reaction
PEA	Palmitoylethanolamide
PPARα	Proliferator-activated receptor alpha
PTV	Policlinico Tor Vergata
ROS	Reactive oxygen species
rRT	reverse transcriptase real time
SARS-CoV-2	Severe acute respiratory syndrome coronavirus-2
TLR	Toll-like receptor
TNF	Tumor necrosis factor
um-PEA	Ultramicronized palmitoylethanolamide
